# *Lactobacillus mucosae* exerted different antiviral effects on respiratory syncytial virus infection in mice

**DOI:** 10.3389/fmicb.2022.1001313

**Published:** 2022-08-26

**Authors:** Qianwen Wang, Zhifeng Fang, Lingzhi Li, Hongchao Wang, Jinlin Zhu, Pinghu Zhang, Yuan-kun Lee, Jianxin Zhao, Hao Zhang, Wenwei Lu, Wei Chen

**Affiliations:** ^1^State Key Laboratory of Food Science and Technology, Jiangnan University, Wuxi, China; ^2^School of Food Science and Technology, Jiangnan University, Wuxi, China; ^3^Institute of Translational Medicine and Jiangsu Key Laboratory of Integrated Traditional Chinese and Western Medicine for Prevention and Treatment of Senile Diseases, Medical College, Yangzhou University, Yangzhou, China; ^4^Department of Microbiology and Immunology, Yong Loo Lin School of Medicine, National University of Singapore, Singapore, Singapore; ^5^International Joint Research Laboratory for Pharmabiotics and Antibiotic Resistance, Jiangnan University, Wuxi, China; ^6^National Engineering Research Center for Functional Food, Jiangnan University, Wuxi, China; ^7^Institute of Food Biotechnology, Jiangnan University, Yangzhou, China; ^8^Wuxi Translational Medicine Research Center and Jiangsu Translational Medicine Research, Institute Wuxi Branch, Wuxi, China

**Keywords:** respiratory syncytial virus, *Lactobacillus mucosae*, immune response, gut microbiota, short-chain fatty acid

## Abstract

Respiratory syncytial virus (RSV) infection is a constant threat to the health of young children, and this is mainly attributed to the lack of effective prevention strategies. This study aimed to determine whether *Lactobacillus* (*L.*) *mucosae*, a potential probiotic, could protect against respiratory viral infection in a mouse model. Naive 3–4-week-old BALB/c mice were orally administered with three *L. mucosae* strains (2.5 × 10^8^ CFU/mouse) 7 days before RSV infection (10^5^ TCID_50_/mouse). Results showed that all three strains inhibited RSV replication and reduced the proportions of inflammatory cells, including granulocytes and monocytes in the blood. The *L. mucosae* M104R01L3 treatment maintained stable weight in mice and increased interferon (IFN)-β and tumor necrosis factor (TNF)-α levels. The *L. mucosae* DCC1HL5 treatment increased interleukin (IL)-1β and IL-10 levels. Moreover, the M104R01L3 and DCC1HL5 strains increased the proportions of *Akkermansia*, *Alistipes*, and *Anaeroplasma* which contributed to the advantageous modulation of the gut microbiota. Besides, *L. mucosae* affected the gut levels of short-chain fatty acids (SCFAs) that are important for the antiviral response. *L. mucosae* 1,025 increased acetate, propionate, and butyrate levels, whereas *L. mucosae* M104R01L3 increased the level of acetate in the gut. *L. mucosae* M104R01L3 may protect against viral infection by upregulating the IFN-β levels in the lungs and its antiviral effect may be related to the increase of acetate levels in the gut. In conclusion, the three *L. mucosae* strains exerted antiviral effects against RSV infection by differentially regulating immune responses and intestinal micro-ecological balance. This study can provide a reference for studying the mechanisms underlying the antiviral effects of *L. mucosae*.

## Introduction

Respiratory syncytial virus (RSV) is an RNA virus of the Paramyxovirus family that causes diseases of variable severity with little systemic symptoms (Openshaw et al., 2017). This virus is responsible for most deaths due to lower respiratory viral infections in young children. In 2015, RSV caused lower respiratory tract infection in approximately 33.1 million children and over 100,000 deaths worldwide (Shi et al., 2017). RSV also threatens the health of individuals who are immunodeficient as a result of aging or lung transplantation (William et al., 2003; Burrows et al., 2015). Although a single RSV infection cannot cause severe disease, compelling evidence suggests that it may result in a secondary bacterial infection that induces more serious lung damage (Damasio et al., 2015). Moreover, RSV reinfection easily occurs because of immune escape even though primary infection induces both humoral and cellular immune responses (Kikkert, 2020). For example, the nonstructural protein NS1 of RSV contributes to immune escape by inhibiting the antiviral type I interferon (IFN) pathway (Hijano et al., 2019). At present, no effective prophylactic measures can protect against RSV infections. Therefore, a safe, universal, and cost-effective prophylactic approach is needed to reduce RSV infections early in life.

The intestinal microbe has recently attracted research interest because it relieves the symptoms of respiratory diseases, including asthma and bacterial and viral infections (Wypych et al., 2019). Previous investigations suggest that the lung health and intestinal micro-ecological balance (Tulic et al., 2016). The intestinal microbiota diversity in mouse models of respiratory viral infection is significantly altered, with increased Bacteroidetes and decreased Firmicutes (Groves et al., 2018) Particularly, RSV infection increases the abundance of *Muribaculaceae*, *Clostridiales*, *Odoribacteraceae*, and *Actinomyces* (Groves et al., 2018, 2020) and reduces that of short-chain fatty acids (SCFA)-producing bacteria, such as *Lachnospiraceae* (Harding et al., 2020). Gut microbes also affect lung health. Viral clearance is delayed, and antiviral immune responses are impaired in mice infected with the influenza virus and treated with antibiotics (Abt et al., 2012). According to present research, there are two main pathways for intestinal microbe to influence lung health. Firstly, toll-like receptors (TLRs) can recognize bacterial components and activate the nuclear factor-kappa B (NF-κB) transcription factor that is a prerequisite for the expression of genes associated with innate immunity and inflammation (Tsay et al., 2011). The intestinal microbe can regulate the toll-like receptors7 signaling pathway to protect against influenza (Wu et al., 2013). Meanwhile, intestinal bacterial metabolites are important to prevent respiratory viral infection. For instance, desaminotyrosine, produced in the gut by *Clostridium orbiscindens*, protects hosts from influenza infection (Steed et al., 2017). SCFAs are common metabolites of the intestinal microbiota that play an important role in protecting against respiratory infections. Influenza infection contributes to bacterial superinfection by reducing the production of SCFAs (Sencio et al., 2020). Especially acetate produced by gut microbiota protects against RSV infection by activating the type I IFN response (Antunes et al., 2019). SCFAs can regulate the balance of T cells and the immune responses (Trompette et al., 2014, 2018).

*Lactobacillus* (*L.*) *mucosae* is a common species of intestinal microbe that produces acetate, and *L. mucosae* 1,025 protects mice against infection with influenza A virus (Lu et al., 2021). Therefore, we hypothesize that *L. mucosae* may have antiviral effects on RSV infection in mice through regulating the gut microbiota and metabolites. The present study explored the antiviral effects of three *L. mucosae* strains in a mouse model infected with the RSV Long strain. The prophylactic effect of the three strains on RSV infection was evaluated by analyzing the clinical symptoms, immune responses, and gut microbiota composition. This study will contribute to explore the mechanisms of *L. mucosae* to protect against RSV infection.

## Materials and methods

### Bacterial strain propagation

*Lactobacillus mucosae* 1,025, *L. mucosae* M104R01L3, and *L. mucosae* DCC1HL5 strains were stored at the Culture Collection of Food Microbiology (CCFM) owned by Jiangnan University. The strains were cultured in De Man, Rogosa, and Sharpe medium (MRS) containing 0.05% (w/v) L-cysteine-HCl at 37°C for 24 h in an anaerobic incubator (AW500SG, Electrotek Scientific Ltd., Shipley, United Kingdom). The mice were orally administered with 2.5 × 10^8^ CFU of the bacterial strain suspension (200 μl).

### Treatments and RSV infection

The animal study was reviewed and approved by the Ethics Committee of Yangzhou University (Approval No. 202011004). Female 3–4-week-old BALB/c mice (Charles River Co., Ltd., Beijing, China) were kept in a facility with a controlled light cycle (12/12 h light/dark), temperature (25 ± 2°C), and humidity level (50%). Mice were fed standard commercial chow and water *ad libitum*. Mice were acclimated for 10 days, then randomly assigned to blank, RSV, positive control, and strain groups (*n* = 8 each).

**Figure 1:**
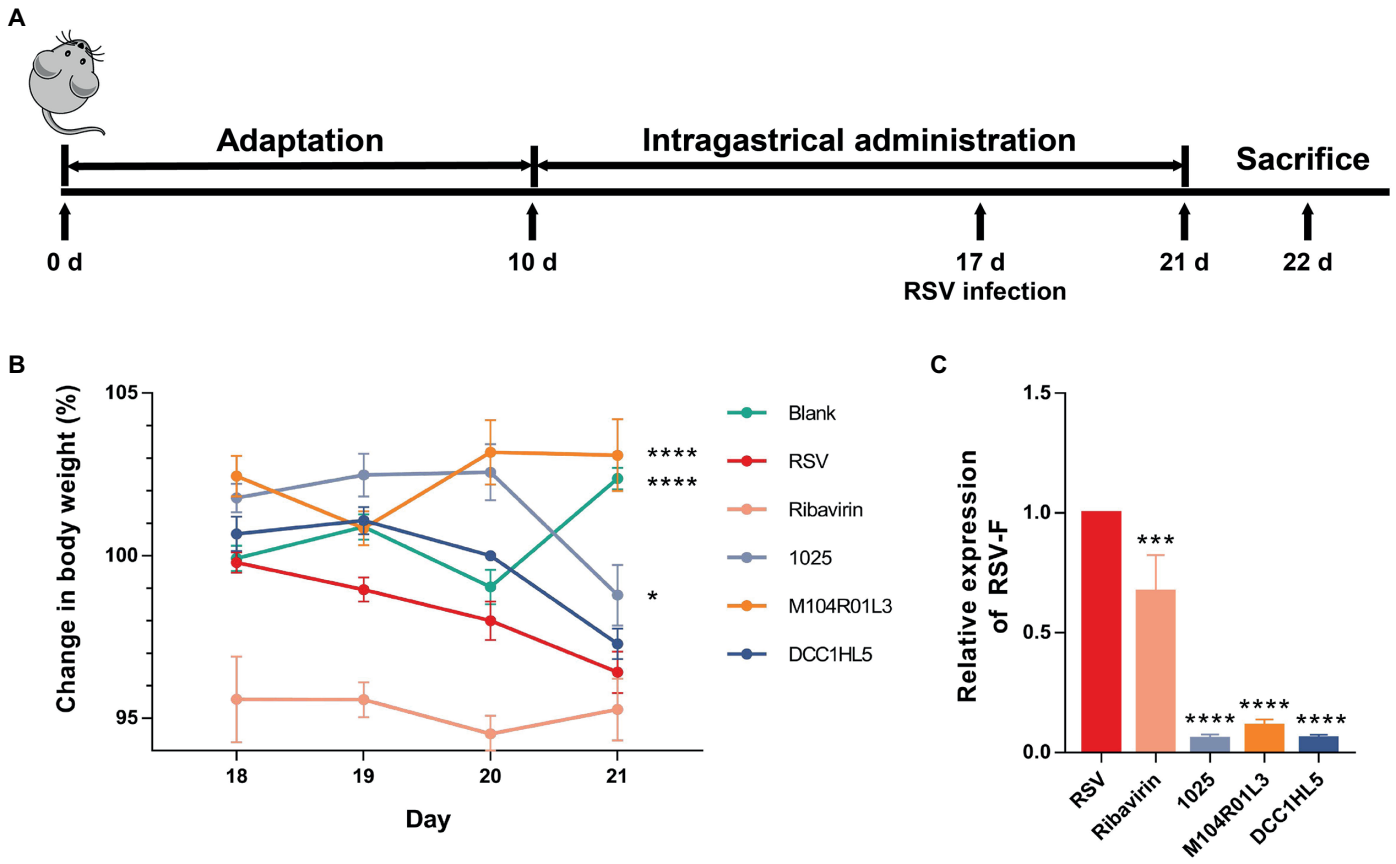
Effects of three *Lactobacillus mucosae* strains on weight loss and viral load in a mouse model of respiratory syncytial virus (RSV) infection. **(A)** Timeline of administering three *L. mucosae* strains to mouse models and infecting them with RSV. **(B)** Changes in body weight of mice. The calculation of the weight change of each mouse was taken their weight of Day 17 as the baseline. **(C)** Viral load in lung tissues (*n* = 8; ^*^p < 0.05, ^***^p < 0.001, ^****^p < 0.0001 vs. RSV group). Error bars indicate the means ± standard error of the mean (SEM). Differences were compared using one-way ANOVA, followed by Fisher’s LSD tests. *P*-values were adjusted using a false discovery rate. Blank, not infected with RSV. RSV, infected with respiratory syncytial virus and untreated.

Animal model of mice with RSV infection was established by intranasal instillation of 10^5^ TCID_50_ RSV Long strain (Key Laboratory of Avian Infectious Diseases, Yangzhou University, Yangzhou, China), and mice achieved a weight loss more than 3% of body weight in 5 days post-infection (Antunes et al., 2019). For 1 week before infection, all mice in the three bacterial strain groups were administered with 2.5 × 10^8^ CFU of bacterial suspension (200 μl) daily (Kei et al., 2019). The blank and RSV groups were administered with an equal volume of sterile saline. All groups except the blank were intranasally infected with RSV on day 17. After infection, mice were continually administered with 200 μl of bacterial suspension or sterile saline for 5 days, until sacrificed after anesthesia on day 22 (Figure 1A). The positive controls were intraperitoneally injected with ribavirin on the day after infection. The mice were weighed daily after RSV infection to assess changes.

### Routine blood analysis

Blood was collected from the sacrificed mice into anticoagulation tubes containing ethylenediaminetetraacetic acid (EDTA)-K2 at room temperature. Blood samples were evaluated using a BC-5000 Vet automated hematology analyzer (Shenzhen Mindray Biomedical Electronics Co., Ltd., Shenzhen, China).

### Protein content in bronchoalveolar lavage fluid

Mouse lungs were lavaged three times with 0.8 ml phosphate-buffered saline to obtain bronchoalveolar lavage fluid (BALF). The protein content in BALF was measured using Enhanced BCA Protein Assay Kits (Beyotime Biotechnology Co., Ltd., Shanghai China).

### Histopathological analysis of lung tissue

The left lobes of the lungs were fixed in 4% paraformaldehyde and embedded in paraffin blocks that were cut into 5-μm sections and stained with hematoxylin and eosin (HE). The stained sections were visualized using a Pannoramic MIDI digital scanner (3DHistech Ltd., Budapest, Hungary). The degree of histological damage to the lungs of four mice per group was scored on a scale of 0–3 (Jeffrey et al., 1991).

### Viral load

Total RNA was extracted from lung tissues using the FastPure® Cell/Tissue Total RNA Isolation Kit (Vazyme Biotech Co., Ltd., Nanjing, China). First-strand cDNA was synthesized from total RNA using HiScript^®^ III SuperMix for qPCR (Vazyme Biotech Co., Ltd., Nanjing, China). Real-time quantitative reverse transcription polymerase chain reaction (qRT-PCR) analysis was performed using iTaq Master SYBR Green Super Mix (Bio-Rad Laboratories Inc., Hercules, CA, United States) and a CFX96 Thermal Cycler (Bio-Rad Laboratories Inc.). The relative expression of genes was normalized to that of GAPDH and calculated using the 2^−ΔΔCT^ method (Livak and Schmittgen, 2001). Table 1 shows the primer sequences.

**Table 1:** Primer sequences for qRT-PCR.

Primers	Forward/reverse	Sequence (5′ to 3′)
GAPDH	Forward	AATGGTGAAGGTCGGTGTGAAC
	Reverse	GCCTTGACTGTGCCGTTGAA
RSV-F	Forward	GCAACCAACAATCGAGCCAG
	Reverse	GGCGATTGCAGATCCAACAC

### Analysis of cytokine concentrations

Lung tissues were homogenized in RIPA Lysis Buffer (200 μl/20 mg tissue; Beyotime Biotechnology Co., Ltd., Shanghai China) containing a protease and phosphatase inhibitor cocktail (Beyotime Biotechnology Co., Ltd., Shanghai China). The homogenates were clarified using centrifugation at 4°C, and the cytokines IFN-β, tumor necrosis factor (TNF)-α, interleukin (IL)-1β, and IL-10 were quantified using the respective ELISA kits (SenBeiJia Co., Ltd., Nanjing, China). Protein results are expressed as BSA equivalents.

### Gut microbial profiling

Fecal samples were collected using sterile tubes on day 21. DNA was extracted from fecal samples (frozen at −80°C) using the Fast DNA SPIN Kit for Feces (MP Biomedicals; Carlsbad, CA, United States), and the 16S rRNA gene was amplified *via* PCR using the specific primers (341F and 806R) for bacterial rRNA. Table 2 shows the primer sequences. The PCR products were purified using DNA Gel/PCR Purification Miniprep Kits (Beiwo Meditech Co., Ltd., Hangzhou, China). Libraries were constructed using a Library Prep Kit for Illumina (Illumina, San Diego, CA, United States) as described by the manufacturer. Index-coded samples were clustered on a cBot Cluster Generation System (Illumina) as described by the manufacturer. The libraries were sequenced using an Illumina MiSeq high-flux sequencing platform (Illumina), and paired-end reads were generated. Finally, *16S* rRNA sequence data were processed as previously described (Caporaso et al., 2010). Bioinformatics analyses included community diversity profiles and taxonomic differences between microbial communities.

**Table 2:** Primer sequences for *16S* rRNA.

Primers	Forward/reverse	Sequence (5′ to 3′)
341F	Forward	CCTAYGGGRBGCASCAG
806R	Reverse	GGACTACNNGGGTATCTAAT

### Analysis of SCFA

SCFAs levels in cecal contents were measured using a GCMS-QP2010 Ultra gas chromatograph-mass spectrometer (Shimadzu Corp., Kyoto, Japan) according to a previously described external standard method (Moreau et al., 2003). Briefly, after acidification with sulfuric acid, SCFAs were extracted with ether and dehydrated with anhydrous sodium sulfate. The supernatant was analyzed using gas chromatography–mass spectrometry (GC–MS).

### Statistical analysis

Differences were compared using one-way analysis of variance (ANOVA), followed by Fisher’s least significant difference (LSD) tests. *p* values were adjusted using the false discovery rate. The error bars of the data indicate the means ± standard error of the mean (SEM). Gut microbial analysis was performed at online web.^1^

## Results

### *Lactobacillus mucosae* exerted antiviral effects on RSV infection

After infection, body weight of mice in the RSV group significantly decreased, whereas *L. mucosae* M104R01L3 and 1,025 strains improved weight loss (Figure 1B). Particularly, *L. mucosae* M104R01L3 maintained stable weight in mice. And the impact of DCC1HL5 treatment on body weight was limited. The positive controls injected with ribavirin weighed less than the RSV group throughout the infection period. Moreover, all *L. mucosae* strains significantly (*p* < 0.0001) suppressed the viral load (expressed relative to levels of the RSV F protein) in lung tissue homogenates on day 5 post-infection and were more effective than ribavirin therapy (Figure 1C). These results indicated that all three *L. mucosae* improved RSV infection, particularly the M104R01L3 strain, which maintained body weight.

### *Lactobacillus mucosae* exerted anti-inflammatory effect on systemic inflammatory responses

**Figure 2:**
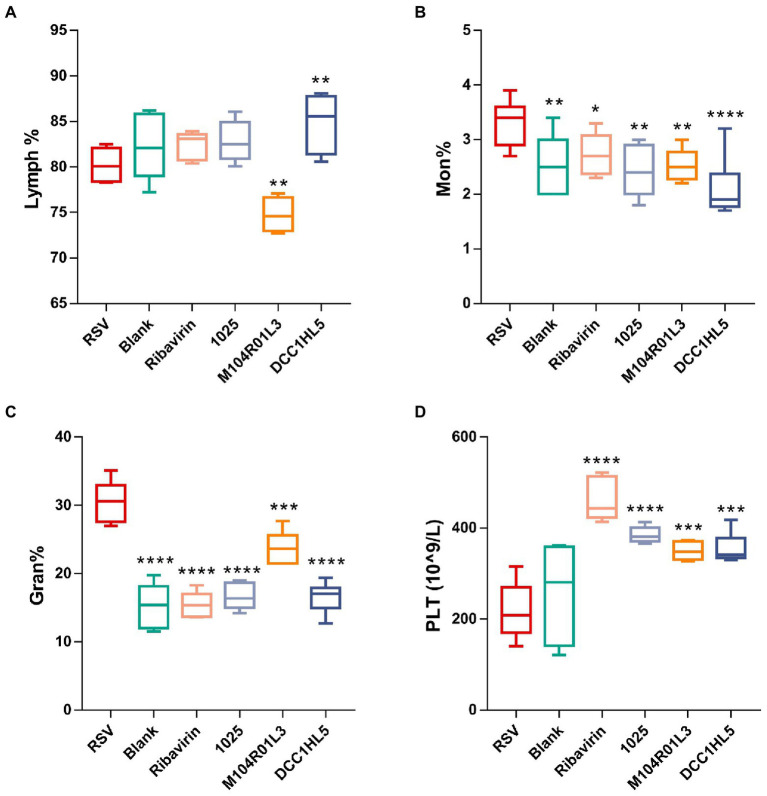
Changes in systemic immune responses. Proportions of **(A)** lymphocytes (Lymph%), **(B)** monocytes (Mon%), and **(C)** granulocytes (Gran%). **(D)** Platelets (PLTs) in blood (*n* = 8; ^*^p < 0.05, ^**^p < 0.01, ^***^p < 0.001, ^****^p < 0.0001 vs. RSV group). Differences were compared using one-way ANOVA, followed by Fisher’s LSD test. *P*-values were adjusted using false discovery rate. Blank, not infected with RSV. RSV, infected with respiratory syncytial virus and untreated.

The effects of *L. mucosae* on systemic inflammatory responses were explored by quantifying changes in blood cell levels *via* routine blood tests. There were no significant changes in the proportion of lymphocytes (lymph%) between the RSV group and the blank group (Figure 2A). The number of lymphocytes was significantly (*p* < 0.01) increased in the DCC1HL5 group (Figure 2A), whereas the M104R01L3 treatment significantly (*p* < 0.01) reduced the lymph%. RSV infection significantly (*p* < 0.01) increased the proportion of monocytes (mon%) in the RSV group. However, the mon% were decreased in the positive control and *L. mucosae* groups, compared with RSV group (Figure 2B). The proportion of neutrophilic granulocytes (gran%) was higher in the RSV group. Like ribavirin in the positive controls, three *L. mucosae* treatments reduced the gran%. It is gratifying that *L. mucosae* 1,025 and DCC1HL5 treatment recovered gran% to normal (Figure 2C). RSV infection did not affect platelet (PLT) counts in the blood, whereas counts increased in the positive control and *L. mucosae* groups (Figure 2D).

Overall, *L. mucosae* strains recovered blood levels of granulocytes and monocytes but increased the numbers of PLTs. *L. mucosae* M104R01L3 treatment significantly reduced the lymphocyte proportions in the blood.

### *Lactobacillus mucosae* exerted different effects on damage and inflammation in the lungs

The extent of the damage was expressed using total protein in BALF. And the effects of *L. mucosae* on lung inflammation were explored by quantifying cytokines and histologically assessing HE-stained lung sections.

**Figure 3:**
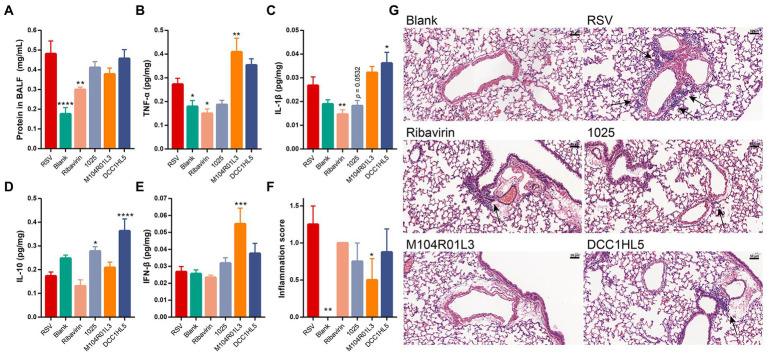
Impact of three *Lactobacillus mucosae* strains on cytokine levels and lung damage. Levels of **(A)** total protein in BALF, **(B)** TNF-α in lung tissues, **(C)** IL-1β in lung tissues, **(D)** IL-10 in lung tissues, and **(E)** IFN-β in lung tissues. **(F)** Inflammation scores of lung tissues (*n* = 4). **(G)** Lung sections stained with hematoxylin and eosin. Arrows, perivascular inflammatory infiltration. Original magnification ×400. (*n* = 8, except (F); ^*^p < 0.05, ^**^p < 0.01, ^***^p < 0.001, ^****^p < 0.0001 vs. RSV group). Error bars show the means ± standard error of the mean (SEM). Differences were compared using one-way ANOVA, followed by Fisher’s LSD test. *P*-values were adjusted using a false discovery rate. Blank, not infected with RSV. RSV, infected with respiratory syncytial virus and untreated.

The levels of total protein were significantly higher in BALF from the RSV group than the blank group (*p* < 0.0001), indicating lung damage in the infected mice. The protein content in BALF was significantly decreased in the positive control group (*p* < 0.01), whereas *L. mucosae* did not improve this index (Figure 3A).

TNF-α and IL-1β are the pro-inflammatory cytokines. RSV infection increased their expression in the lungs. *L. mucosae* regulated the expression of these cytokines in lung tissues. The *L. mucosae* 1,025 treatment exhibited a downward trend in the levels of pro-inflammatory cytokines, whereas the M104R01L3 treatment increased the levels of TNF-α and the DCC1HL5 treatment increased the levels of IL-1β in the lung tissues. However, ribavirin noticeably downregulated the expression of these pro-inflammatory cytokines (Figures 3B,C). IL-10 is an anti-inflammatory cytokine. RSV infection did not affect the levels of this cytokine. The concentrations of IL-10 were significantly increased in the 1,025 and DCC1HL5 groups (Figure 3D).

The levels of the major antiviral mediator, IFN-β, were in quantified lung homogenates. RSV infection did not activate the IFN-β response or change its concentration compared with that of the blank group. *L. mucosae* strains increased the levels of IFN-β. Particularly, this cytokine was significantly increased in the M104R01L3 group (Figure 3E).

Histological section is an important indicator to assess the damage and inflammation in the lungs. The sections histologically assessed the effects of *L. mucosae* treatments on pathological lung symptoms. The results showed perivascular infiltration of inflammatory cells after RSV infection compared with the blank. However, *L. mucosae* alleviated the inflammatory infiltration induced by viral infection. In particular, the inflammation score was lower for the M104R01L3 group than that of the 1,025 and DCC1HL5 groups (Figures 3F,G).

Hence, *L. mucosae* exerted different effects on host inflammation in the lungs. *L. mucosae* 1,025 strain reduced the expression of the pro-inflammatory cytokines and increased the levels of the anti-inflammatory cytokine. Specifically, IFN-β levels increased in the M104R01L3 group. According to histological sections and inflammation score, *L. mucosae* M104R01L3 exhibited the best improvement in the lungs.

### *Lactobacillus mucosae* altered the gut microbiota composition

**Figure 4:**
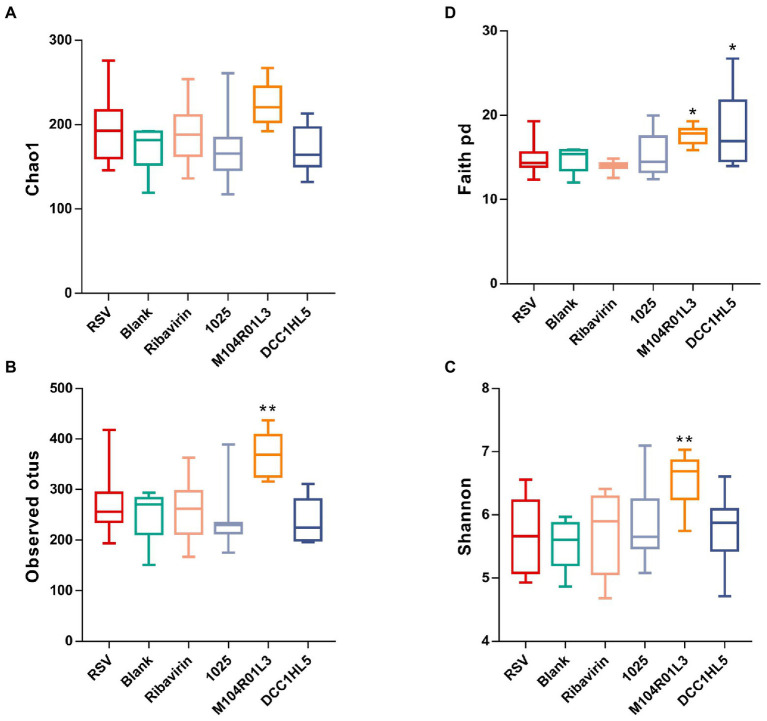
Impact of three *Lactobacillus mucosae* strains on the alpha-diversity of the gut microbiota. **(A)** Chao1, **(B)** observed operational taxonomic units (OTUs), **(C)** Shannon, and **(D)** Faith’s phylogenetic diversity indices. (*n* = 8; ^*^p < 0.05, ^**^p < 0.01 vs. RSV group). Differences were compared using one-way ANOVA, followed by Fisher’s LSD test. p values were adjusted using a false discovery rate. Blank, not infected with RSV. RSV, infected with respiratory syncytial virus and untreated.

Alpha-diversity indices were analyzed to determine the diversity of the gut microbiota among the groups. The Chao1 index did not significantly differ among the groups (Figure 4A). According to the Shannon and observed operational taxonomic unit (OTU) indices (Figures 4B,C), RSV infection did not obviously change the diversity of the gut microbiota. However, these indices were significantly (*p* < 0.01) increased in the M104R01L3 group but not in the other groups. The Faith’s phylogenetic diversity (faith_pd) index of the M104R01L3 and DCC1HL5 groups was significantly higher than that of the RSV group (Figure 4D).

According to the beta-diversity analysis, RSV infection minimally affected the gut microbiota diversity, whereas *L. mucosae* altered the beta diversity of the gut microbiota (Supplementary Figure 1). The composition of the gut microbiota was analyzed at the phylum level to determine changes. The relative abundance of Bacteroidetes was higher in the 1,025 and M104R01L3 groups (Supplementary Figure 2A), whereas M104R01L3 treatment decreased the abundance of Firmicutes (Supplementary Figure 2B), and *L. mucosae* treatments had no effect on the abundance of Proteobacteria (Supplementary Figure 2C). DCC1HL5 treatment increased the relative abundance of the Verrucomicrobia family (Supplementary Figure 2D). Actinobacteria were more abundant in the RSV group than that of the blank group (Supplementary Figure 2E). The *L. mucosae* strains exerted different effects on gut microbiota composition at the family level using linear discriminant analysis effect size (LEfSe) analysis (Supplementary Figure 3A). At the family level, all of them increased the relative abundance of *Erysipelotrichaceae* (Supplementary Figures 3C–E). The abundance of *Muribaculaceae* (S24-7) was significantly higher in the 1,025 and M104R01L3 groups (Supplementary Figures 3C,D). The relative abundance of *Akkermansiaceae* was increased in the DCC1HL5 group (Supplementary Figure 3E).

**Figure 5:**
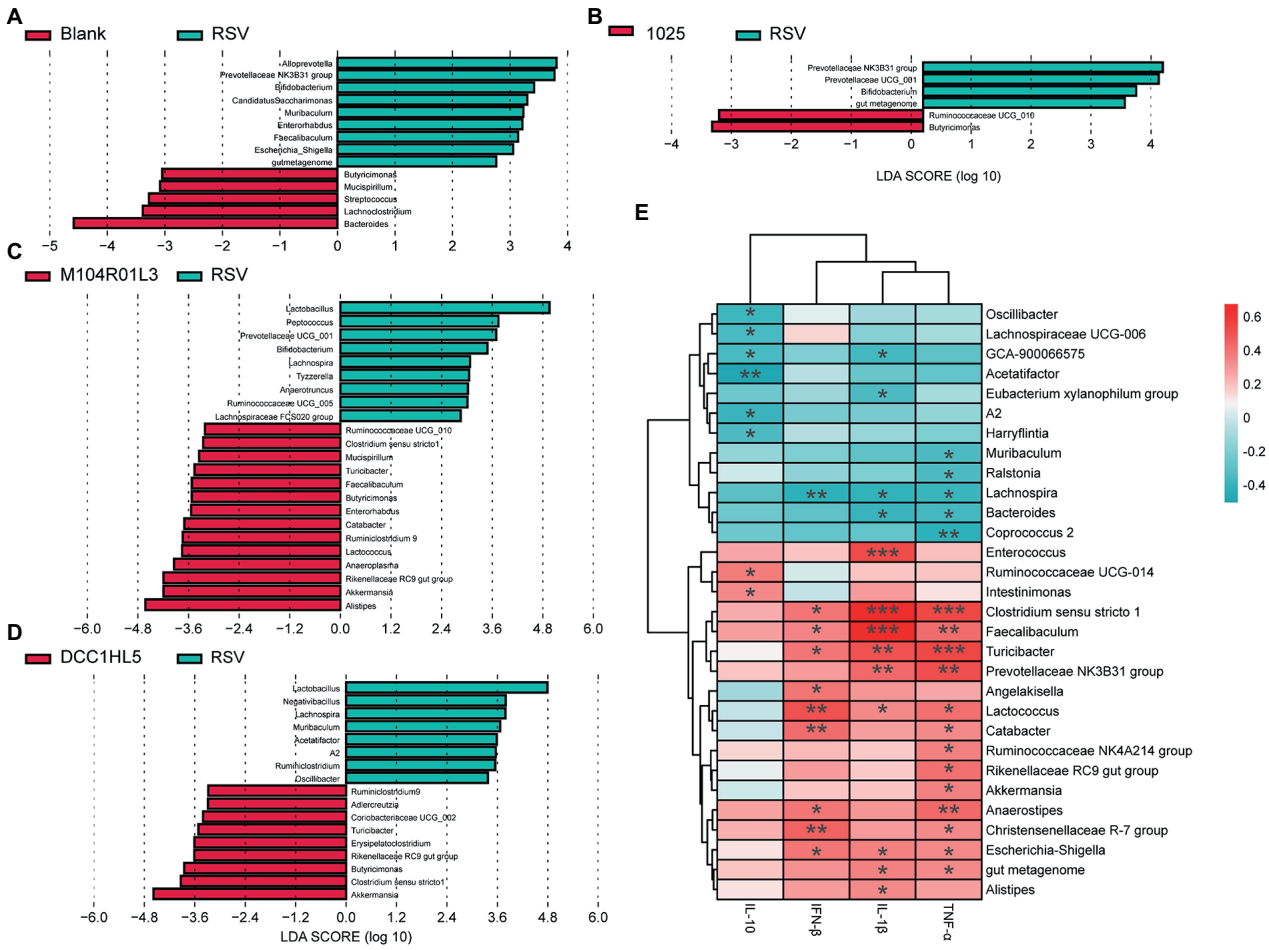
Impact of three *Lactobacillus mucosae* strains on the relative abundance of bacterial genera and correlation analysis. Linear discriminant analysis effect size (LEfSe) comparison of gut microbes at the genus level between **(A)** blank and RSV groups, **(B)** 1,025 and RSV groups, **(C)** M104R01L3 and RSV groups, and **(D)** DCC1HL5 and RSV groups. **(E)** Correlations between cytokines and gut microbiota. (*n* = 8; ^*^p < 0.05, ^**^p < 0.01, ^***^p < 0.001). Blank, not infected RSV. RSV, infected with respiratory syncytial virus and untreated.

The composition of the gut microbiota was analyzed at the genus level using LEfSe analysis. RSV infection altered the relative abundance of genera associated with SCFA production, including decreased *Lachnoclostridium* and *Butyricimonas*. And the abundances of *Alloprevotella* and *Prevotellaceae* NK3B31 group were higher than the blank group (Figure 5A). However, all *L. mucosae* groups increased the abundance of *Butyricimonas*, compared with RSV group (Figures 5B–D). Except for *Butyricimonas*, the *L. mucosae* 1,025 group had a higher abundance of *Ruminococcaceae* UCG-010, as well as that in the *L. mucosae* M104R01L3 group (Figures 5B,C). *L. mucosae* 1,025 treatment specially reduced the levels of *Prevotellaceae* NK3B31 group (Figure 5B). Furthermore, M104R01L3 and DCC1HL5 strains exerted similar effects on gut microbiota composition; they increased the relative abundance of *Akkermansia*, *Clostridium sensu stricto* 1, *Rikenellaceae* RC9, *Turicibacter*, and *Ruminiclostridium* 9. Notably, the DCC1HL5 strain enriched the gut microbiota of mice with *Adlercreutzia* spp. The M104R01L3 treatment resulted in a higher abundance of *Alistipes* and Anaeroplasma (Figures 5C,D). These results indicated that *L. mucosae* strains modulated gut microbiota differently at the genus level.

Correlation analysis were performed between the gut microbiota and cytokines, using Spearman’s rank correlation coefficient, to determine the impact of the gut microbiota on host inflammatory cytokines to RSV infection. The levels of TNF-α, IL-1β, and IFN-β were positively correlated with the abundances of *Clostridium sensu stricto* 1, *Faecalibaculum*, *Turicibacter*, *Lactococcus*, and *Escherichia-Shigella*. The abundances of *Prevotellaceae* NK3B31 were positively correlated with the levels of TNF-α and IL-1β. However, the abundances of *Lachnospira* and *Bacteroides* were negatively correlated with the levels of these pro-inflammatory cytokines. In addition, the abundances of *Catabacter*, *Akkermansia*, and *Rikenellaceae* RC9 were positively associated with increased TNF-α expression. The abundances of *Enterococcus* and *Alistipes* were positively correlated with the levels of IL-1β. And the TNF-α levels were negatively correlated with the levels of *Muribaculum*, *Ralstonia*, and *Coprococcus* 2. In particular, the abundances of *Angelakisella* were positively correlated with the IFN-β level and the abundances of *Lachnospira* were negatively correlated with this cytokine. The IL-10 levels were positively correlated with *Ruminococcaceae* UCG-014 and *Intestinimonas*, while it was negatively correlated with *Oscillibacter*, *Lachnospiraceae* UCG-006, *Acetatifactor*, A2, and *Harryflintia* (Figure 5E). To sum up, the upregulations of pro-inflammatory cytokines were positively correlated with the abundances of *Clostridium sensu stricto* 1, *Faecalibaculum*, *Turicibacter*, *Prevotellaceae* NK2B31, *Lactococcus*, *Catabacter*, *Akkermansia*, *Rikenellaceae* RC9, *Enterococcus*, and *Alistipes*. And the abundances of *Lachnospira*, *Bacteroides*, *Muribaculum*, *Ralstonia*, and *Coprococcus* 2 were negatively correlated with the levels of these cytokines. The levels of anti-inflammatory cytokine were positively correlated with *Ruminococcaceae* UCG-014 and *Intestinimonas*; negatively correlated with *Oscillibacter*, *Lachnospiraceae* UCG-006, *Acetatifactor*, A2, and *Harryflintia*.

### *Lactobacillus mucosae* strains affected SCFA production

**Figure 6:**
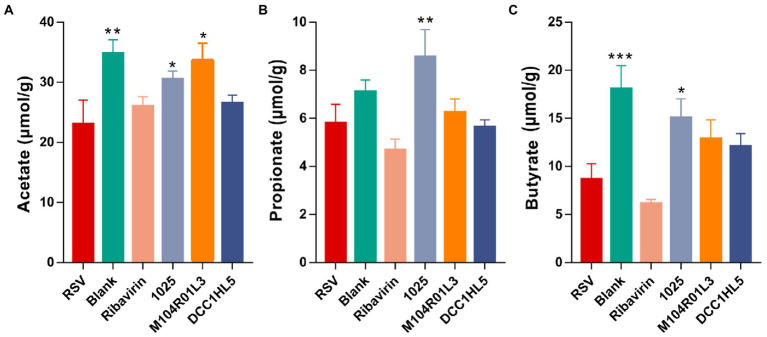
Levels of SCFAs in mouse ceca. **(A)** Acetate. **(B)** Butyrate. **(C)** Propionate (*n* = 8. ^*^p < 0.05, ^**^p < 0.01, ^***^p < 0.001 vs. RSV group). Error bars indicate the means ± standard error of the mean (SEM). Differences were compared using one-way ANOVA followed by Fisher’s LSD test. p values were adjusted using a false discovery rate. Blank, not infected with RSV. RSV, infected with respiratory syncytial virus and untreated.

SCFAs are effective intestinal metabolites for alleviating respiratory diseases, including viral infections (Antunes et al., 2019). The levels of acetate, propionate, and butyrate were measured in cecal contents using GC–MS. RSV infection decreased all the levels of SCFAs, especially acetate and butyrate (Figures 6A–C). The results showed that 1,025 and M104R01L3 strains increased the concentrations of SCFAs, particularly acetate (Figure 6A). Acetate levels were higher in the M104R01L3 group than that in the other groups. And the 1,025 strain significantly increased the levels of propionate (Figure 6B). Furthermore, the 1,025 strain generated the most butyrate among all groups, although other strains also triggered increased butyrate production (Figure 6C). Particularly, 1,025 strains significantly increased all the levels of SCFAs.

## Discussion

Gut microbiota and probiotics exert preventive and alleviating effects on respiratory diseases, including viral and bacterial infections; however, the mechanisms are still not completely understood. According to the gut–lung axis theory, the gut microbiota influences lung health by generating soluble microbial components and metabolites that are transported *via* circulation (Wypych et al., 2019). Therefore, understanding gut microbial alterations that protect against respiratory diseases could contribute to the clinical applications of probiotics. *L. mucosae* modulates immune system tone (Ryan et al., 2019), and strain 1,025 significantly protects against influenza A infection (Lu et al., 2021). The present study investigated the prophylactic effects of three *L. mucosae* strains against infection with RSV Long strain. The results showed that all three strains significantly decreased viral load in the lungs, through different effects on cytokine levels and routine blood parameters. It is indicated that *L. mucosae* have various mechanisms to inhibit RSV replication and exert prophylactic effects against RSV infection.

The antiviral mechanisms of three strains of *L. mucosae* were explored by evaluating IFN-β levels as an indicator of the type I IFN response. Type I IFNs are major antiviral effectors that mediate antiviral responses, including the Mx GTPase pathway and the 2′,5′-oligoadenylate synthetase-directed ribonuclease L pathway (Sadler and Williams, 2008). Proteins of RSV may be an important means of inhibiting antiviral type I IFN expression through multiple pathways, thus promoting viral replication (Hijano et al., 2019). RSV does not induce strong, long-term immunity because of innate immune evasion; this leads to recurrent infections with the same or different strains of RSV (Paul et al., 2006). In the present study, RSV infection did not increase IFN-β expression in the lungs. However, M104R01L3 treatment, but neither 1,025 nor DCC1HL5 treatments, significantly increased IFN-β levels. This indicated that M104R01L3 treatment could activate type I IFN expression in the lungs to inhibit viral replication. Moreover, both M104R01L3 and DCC1HL5 treatments increased the levels of TNF-α in the lungs. This pro-inflammatory cytokine has been regarded as a factor that exacerbates illness. However, TNF-α plays a protective role against RSV infection (Neuzil et al., 1996). It enhances the expression of the TLR and retinoic acid-inducible gene I (RIG-I) signaling pathways, which stimulate more cytokine and chemokine production in lung epithelial cells (Matikainen et al., 2006). Infants infected with RSV have less TNF-α synthesis than healthy infants, implying an impaired early innate immune response (Kreso et al., 2010). Anti-TNF-α treatment leads to a higher RSV lung viral load compared with that in the controls (Groves et al., 2020). According to these results, M104R01L3 and DCC1HL5 treatment may protect against RSV infection through a TNF-α-dependent pathway.

Based on the IL-1β concentrations in the lungs, it was speculated that DCC1HL5 treatment may induce the expression of TNF-α associated with IL-1β, which is a pro-inflammatory cytokine involved in innate immunity. Moreover, IL-1β induces TNF-α-mediated inflammatory responses in lung epithelial cells by improving TNF receptor surface expression in epithelial cells (Saperstein et al., 2009). IL-1β indirectly influences viral clearance by activating the TLR7 signaling pathway (Abdul-Cader et al., 2018). The small hydrophobic (SH) protein of RSV inhibits TNF signaling, and recombinant RSV without an SH gene increases the levels of IL-1β and TNF (Russell et al., 2015). In terms of DCC1HL5 treatment, IL-1β might be a critical factor for regulating immunity and protecting against RSV infection. Probiotics increase the levels of inflammatory cytokines in the lungs when they inhibit RSV replication. A regulated inflammatory response is necessary for pathogen elimination. Probiotics might regulate the expression of pro- and anti-inflammatory factors to balance immune responses. For example, treatment with *L. rhamnosus* CRL1505 induces TNF-α production while increasing IL-10 levels, leading to an effective and safe response against RSV infection (Yohsuke et al., 2013). In this study, DCC1HL5 treatment upregulated IL-10 secretion in the lungs, but not M104R01L3; M104R01L3 treatment might regulate this balance through different pathways. Moreover, the regulation of pro- and anti-inflammatory factors is not synchronous, which unliked *L. rhamnosus* CRL1505. M104R01L3 and DCC1HL5 treatments protect against RSV infection by activating inflammatory responses. In contrast, *L. mucosae* 1,025, a potential anti-inflammatory probiotic, decreased and increased the levels of pro- and anti-inflammatory cytokines, respectively. Surprisingly, *L. mucosae* 1,025 did not induce IFN-β expression. These results suggested that the antiviral mechanism of *L. mucosae* 1,025 is independent of both type I IFN and inflammatory responses. The mechanism by which *L. mucosae* 1,025 inhibits viral replication remains unknown and requires further investigation.

In the present study, RSV infection led to low lymphocyte counts in the blood with no significant difference. RSV infection restrains the development of cytotoxic responses, such as the induction of lymphocyte apoptosis (Roe et al., 2004) and alteration in dendritic cell function (Guerrero-Plata et al., 2006). The mechanism of apoptosis induced by RSV is responsible for the low lymphocyte counts. Lymphopenia during measles infection is thought to induce T cell immunosuppression, like RSV infection (O’Donnell and Carrington, 2002). T cell proliferative responses, the development of CD8+ T cell memory, and CD8+ T cell infiltration might be inhibited by RSV (Chang and Braciale, 2002). The increased lymphocyte levels in the DCC1HL5 group suggested that it might activate the systemic immune response. However, this requires further investigation. Interestingly, M104R01L3 treatment significantly decreased lymphocyte levels, which might be the result of redistribution in the lungs (Smith et al., 2001). Whether M104R01L3 treatment affects lymphocytes in accordance with this hypothesis requires further investigation. Neutrophils are critical effector cells of the innate immune system and are the predominant inflammatory cells recruited to the respiratory tract (Galani and Andreakos, 2015). They are also associated with disease severity in RSV infections (Kozo et al., 2005). Although neutrophils possess various defensive strategies that protect against pathogens, they may also cause collateral damage to host tissue (Cortjens et al., 2016). In the present study, all treatments, except for M104R01L3, observably decreased and recovered the level of neutrophilic granulocytes to the normal level. Appropriate neutrophil apoptosis is important for the resolution of inflammation (Driss and Filep, 2010). Platelets have a variety of transmembrane receptors, including TLRs and TNF receptors, which might be associated with the regulation of immune responses (Zamani-Rarani et al., 2022). During COVID-19, platelets play an important role in the host’s antiviral responses (Trugilho et al., 2022). Interestingly, the levels of blood platelets significantly increased after ribavirin and *L. mucosae* strains treatments. The activation of platelets might contribute to damage repair and increase antiviral responses. Besides, the alteration of platelets might be due to the change in gut microbiota composition (Joseph et al., 2021). Future studies are needed to explore the mechanisms of increasing platelets in *L. mucosae* treatments.

This study indicated that *L. mucosae* can modulate systemic immune responses to alleviate inflammation induced by RSV infection.

The gut microbiota plays a critical role in protecting against pathogen infection through modulating the innate and adaptive immune responses. According to the gut–lung axis theory, RSV infection alters gut microbiota and metabolites. The alpha diversity is lower, and beta diversity significantly differs in the gut microbiota of patients with severe disease after RSV infection compared with that in the controls (Harding et al., 2020). However, RSV infection did not markedly affect either alpha or beta diversity in this study. The M104R01L3 and DCC1HL5 strains changed the composition of the gut microbiota, but *L. mucosae* 1,025 had little effect. Our analysis of the gut microbiota also suggested that the distinct immunoregulatory and antiviral activities mediated by *L. mucosae* during RSV infection might be associated with distinct bacterial genera. The abundance of *Butyricimonas*, which converts carbohydrates to butyrate, plays an important role in maintaining a beneficial gut environment during RSV infection. The three *L. mucosae* strains restored the abundance of *Butyricimonas*. In the *L. mucosae 1,025* group, the abundance of *Butyricimonas* might have been a major factor that resisted RSV infection when the strain supplement also increased the abundance of *Ruminococcaceae* UCG-010. *Butyricimonas* was negatively correlated with the IL-1β and IL-6 levels but positively correlated with those of TNF-α and IL-10 (Lee et al., 2018; An et al., 2021). In this study, the levels of pro-inflammatory cytokines were negatively correlated with the abundances of *Prevotellaceae* NK3B31, which were reduced in the *L. mucosae* 1,025 group compared with the RSV group. The anti-inflammatory mechanism of *L. mucosae* 1,025 may be associated with the enrichment of both *Butyricimonas* and *Ruminococcaceae* UCG-010 and the decrease of *Prevotellaceae* NK3B31. Although the abundances of *Butyricimonas* and *Ruminococcaceae* UCG-010 also increased in the M104R01L3 and DCC1HL5 groups, other genera may play a greater role in modulating inflammation and resisting viral infections. M104R01L3 and DCC1HL5 treatments increased the abundances of *Turicibacter* and *Clostridium sensu stricto* 1, which are considered pro-inflammatory taxa (Wang et al., 2017; Ma et al., 2018). The results of the correlation analysis indicated that enriched *Turicibacter* and *Clostridium sensu stricto* 1 might contribute to the increased levels of IL-1β and TNF-α in the M104R01L3 and DCC1HL5 groups. *Turicibacter* might be associated with bile acid metabolism (Kemis et al., 2019), while RSV infection downregulates the metabolism of primary and secondary bile acids (Groves et al., 2020). In addition, *Akkermansia*, a promising probiotic candidate, increased in M104R01L3 and DCC1HL5 groups. This genus exerted beneficial effects during H7N9 infection (Hu et al., 2020). In the present study, *Akkermansia* might play a beneficial role in protecting mice against RSV infection by inducing TNF-α expression. These results suggested that these strains might help the host fight off virus infection by increasing the abundance of *Akkermansia*. Except these genera, M104R01L3 characteristically increased the abundances of *Alistipes* and *Anaeroplasma*, which are prime candidates for effective anti-inflammatory probiotics (Beller et al., 2020; Parker et al., 2020). The increase of *Alistipes* might be associated with the increased levels of blood platelets (Joseph et al., 2021). In addition, *Faecalibaculum*, *Lactococcus*, and *Catabacter* were increased in the M104R01L3 group, which might have contributed to the upregulated IFN-β. The DCC1HL5 treatment increased the abundance of *Adlercreutzia*, an equol-producing bacteria (Bian et al., 2017). *Adlercreutzia* and its metabolites can increase anti-inflammatory capacity in the host (Wei et al., 2018). Our results suggested that *L. mucosae* protected the host from RSV infection associated with alteration of the gut microbiota composition.

The host interacts with microbiota-derived metabolites that are important for protection against pathogens. SCFAs, produced by the gut microbiota, regulate immune and antiviral responses that protect the host against respiratory infections. In the present study, RSV infection reduced the levels of SCFAs, especially acetate and butyrate. *L. mucosae* treatment significantly increased SCFA concentrations after RSV infection. Combined with the gut microbiota findings, the modulation of SCFA-producing bacteria contributed to the changes in cecal SCFAs. Unmetabolized SCFAs enter the lungs *via* the peripheral circulation (Wypych et al., 2019; Sencio et al., 2020). The antiviral mechanisms of the type I IFN response in lung tissues may be overcome by SCFAs in different ways. Probiotics protect against RSV-induced pathology through IFN-β derived from alveolar macrophages, which is attributed to increased acetate level (Ji et al., 2021). Acetate also exerts its effects on epithelial cells (Antunes et al., 2019). In addition to acetate, butyrate can induce a type I IFN response (Patel et al., 2012), and this correlates with the production of desaminotyrosine (Bastiaan et al., 2018), which is associated with the activation of type I IFN signaling that ameliorates influenza infection (Steed et al., 2017). Based on the upregulation of IFN-β expression in lung tissues, M104R01L3 treatment might recover the type I IFN response to protect against RSV infection by increasing gut levels of acetate. Furthermore, SCFAs also enter the bone marrow, which is the main site of immune cell development (Dang and Marsland, 2019). As RSV evades the adaptive immune response by skewing the balance of T helper type 1 (Th1)/Th2 toward a Th2-specific immune response (Becker, 2006), recovering the balance of T cells might inhibit RSV replication. Circulatory SCFAs can modulate dendritic cell hematopoiesis and functionality in the bone marrow and impair Th2 differentiation (Trompette et al., 2014). Moreover, SCFAs improve the host response to influenza infection by dampening deleterious neutrophil-dependent immunopathology with antiviral CD8+ T cell responses enhanced by increasing T cell metabolism. SCFAs can prevent neutrophil influx into the airways by retaining them in the bone marrow (Trompette et al., 2018). Based on these previous studies, this research indicates that *L. mucosae* may restore SCFAs levels to decrease neutrophil levels in the blood. Besides, Butyrate and propionate induce peripheral forkhead box protein P3 (FoxP3+) regulatory T cells, which promote influenza-specific T follicular helper. Butyrate is an immune-suppressant *via* reducing the expression of co-stimulatory surface molecules and impairs T cell activation (Stricker et al., 2022). And the increased levels of SCFAs might inhibit the production of pro-inflammatory cytokines (Kruk et al., 2022). In the *L. mucosae* 1,025 group, butyrate and propionate might contribute to the anti-inflammatory and antiviral effects (Anderson and Reiter, 2020).

According to the gut–lung axis theory, *L. mucosae* M104R01L3 might activate the type I IFN responses by increasing the levels of acetate in the gut to inhibit the virus replication. The microbiota-derived acetate might activate the antiviral activity in the pulmonary epithelial cells *via* the peripheral circulation. *L. mucosae* 1,025 might decrease the inflammation by increasing the levels of butyrate and propionate that were transported *via* circulation. However, L. mucosae DCC1HL5 exerted fewer effects on SCFAs levels, its mechanisms of protecting against RSV infection might associated with the increase of *Turicibacter* and *Clostridium sensu stricto* 1 in the gut.

## Conclusion

Three *L. mucosae* strains exerted antiviral effects on RSV infection by regulating the host immune responses and gut microbiota composition. Among the three strains, *L. mucosae* 1,025 treatment exhibited anti-inflammatory effects during RSV infection. M104R01L3 treatment induced the type I IFN response to protect against viral infection, whereas DCC1HL5 regulated the balance between anti- and pro-inflammatory cytokine levels. This study showed that *L. mucosae* prevent respiratory viral infection *via* various mechanisms, and gut microbiota and metabolites play essential roles. The findings of the present study can contribute to the future development of probiotics as prophylactic agents for RSV infections.

## Data availability statement

The datasets presented in this study can be found in online repositories. The names of the repository/repositories and accession number(s) can be found at: https://www.ncbi.nlm.nih.gov/, PRJNA861107.

## Ethics statement

The animal study was reviewed and approved by the Ethics Committee of Yangzhou University.

## Author contributions

QW and WL: conceptualization. QW, WL, LL, and ZF: methodology. ZF and QW: software. QW, ZF, LL, and WL: validation. QW, HW, and JZhu: formal analysis. QW: investigation and writing—review and editing. JZha and WC: resources. QW, Y-kL, and JZhu: data curation. QW and LL: writing—original draft preparation. QW, ZF, and Y-kL: visualization. PZ, WL, and WC: supervision. PZ and WL: project administration. HZ and WC: funding acquisition. All authors contributed to the article and approved the submitted version.

## Funding

This research was supported by the National Natural Science Foundation of China (Grant No. 31820103010 and 32021005) and 111 Project (no. BP0719028).

## Conflict of interest

The authors declare that the research was conducted in the absence of any commercial or financial relationships that could be construed as a potential conflict of interest.

## Publisher’s note

All claims expressed in this article are solely those of the authors and do not necessarily represent those of their affiliated organizations, or those of the publisher, the editors and the reviewers. Any product that may be evaluated in this article, or claim that may be made by its manufacturer, is not guaranteed or endorsed by the publisher.

## Footnotes

^1^ https://www.bioincloud.tech/
