# Supplementary material for: Lactobacillus mucosae exerted different antiviral effects on respiratory syncytial virus infection in mice
Source: Front Microbiol. 2022 Aug 26;13:1001313. doi: 10.3389/fmicb.2022.1001313 (PMC9459143; doi:10.3389/fmicb.2022.1001313)
Supplement: Supplementary file 1 [file Data_Sheet_1.docx]

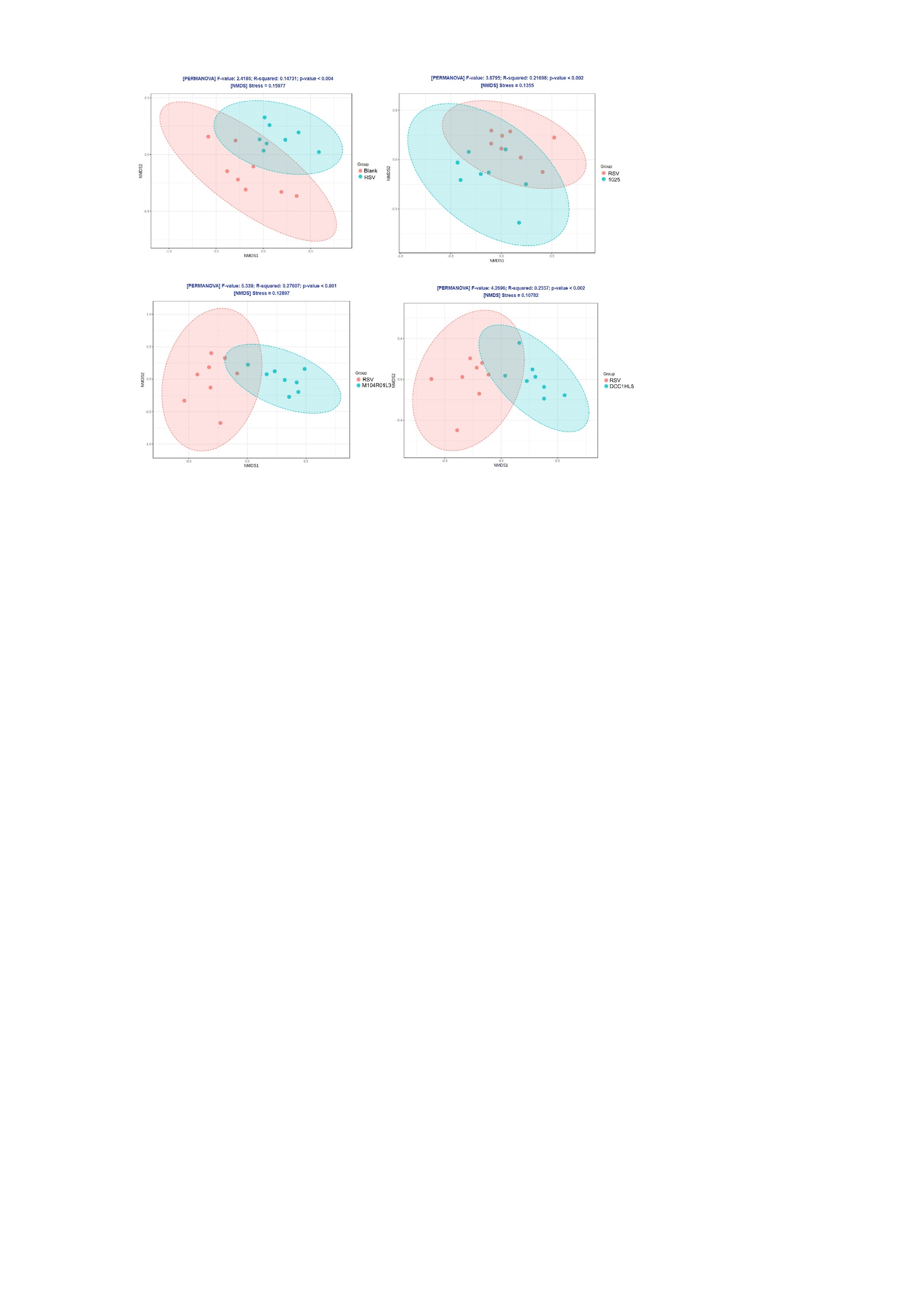


**Supplementary Figure 1|** Impact of three *L. mucosae* strains on beta diversity of gut microbiota at the genus level.


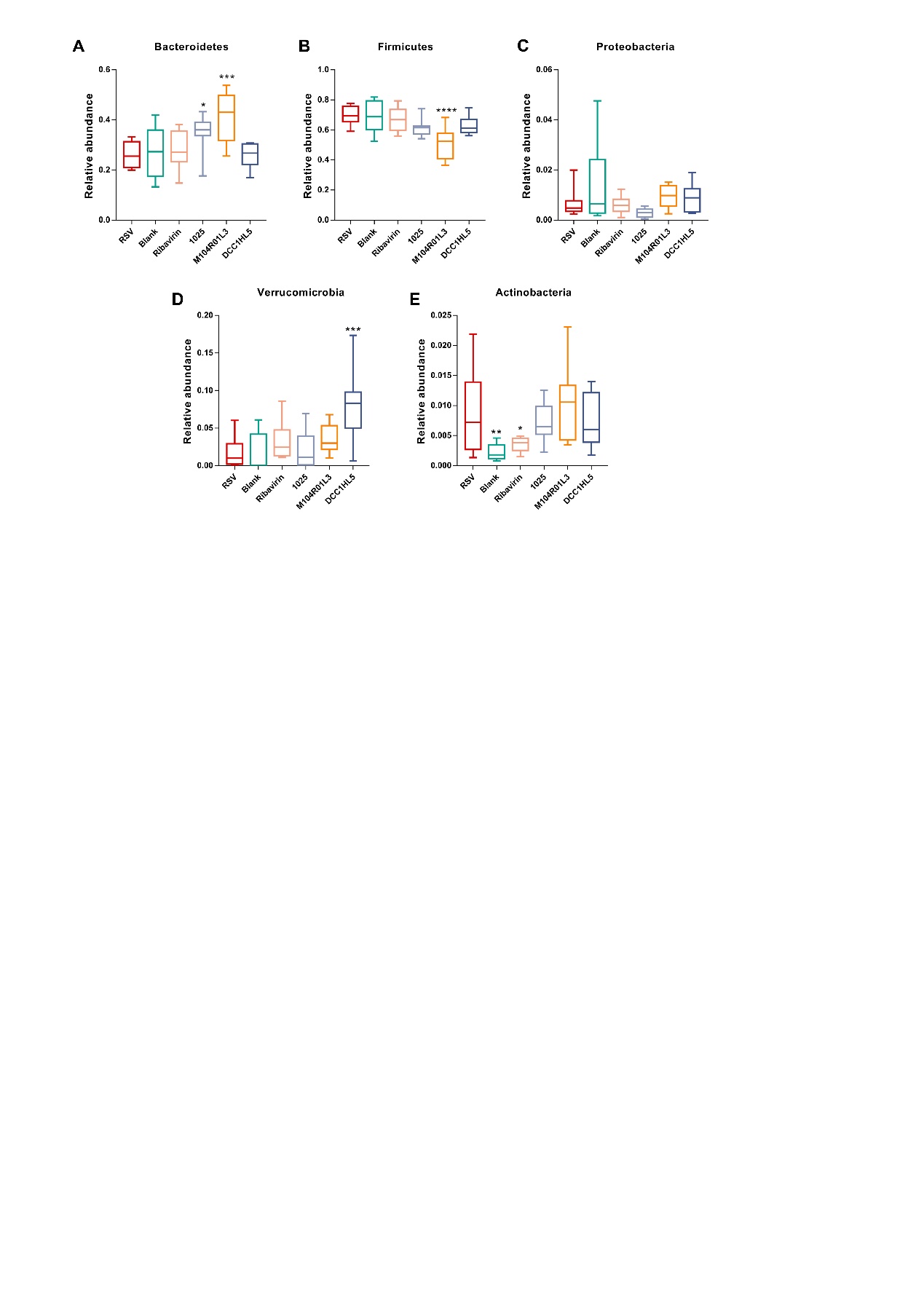


**Supplementary Figure 2|** Impact of three *L. mucosae* strains on the relative abundance of bacterial phyla.
Relative abundance of **(A)** Bacteroidetes, **(B)** Firmicutes, **(C)** Proteobacteria, **(D)** Verrucomicrobia. and **(E)** Actinobacteria. Differences were compared using one-way ANOVA, followed by Fisher’s LSD test. P values were adjusted using a false discovery rate. Blank, not infected with RSV. RSV, infected with respiratory syncytial virus and untreated


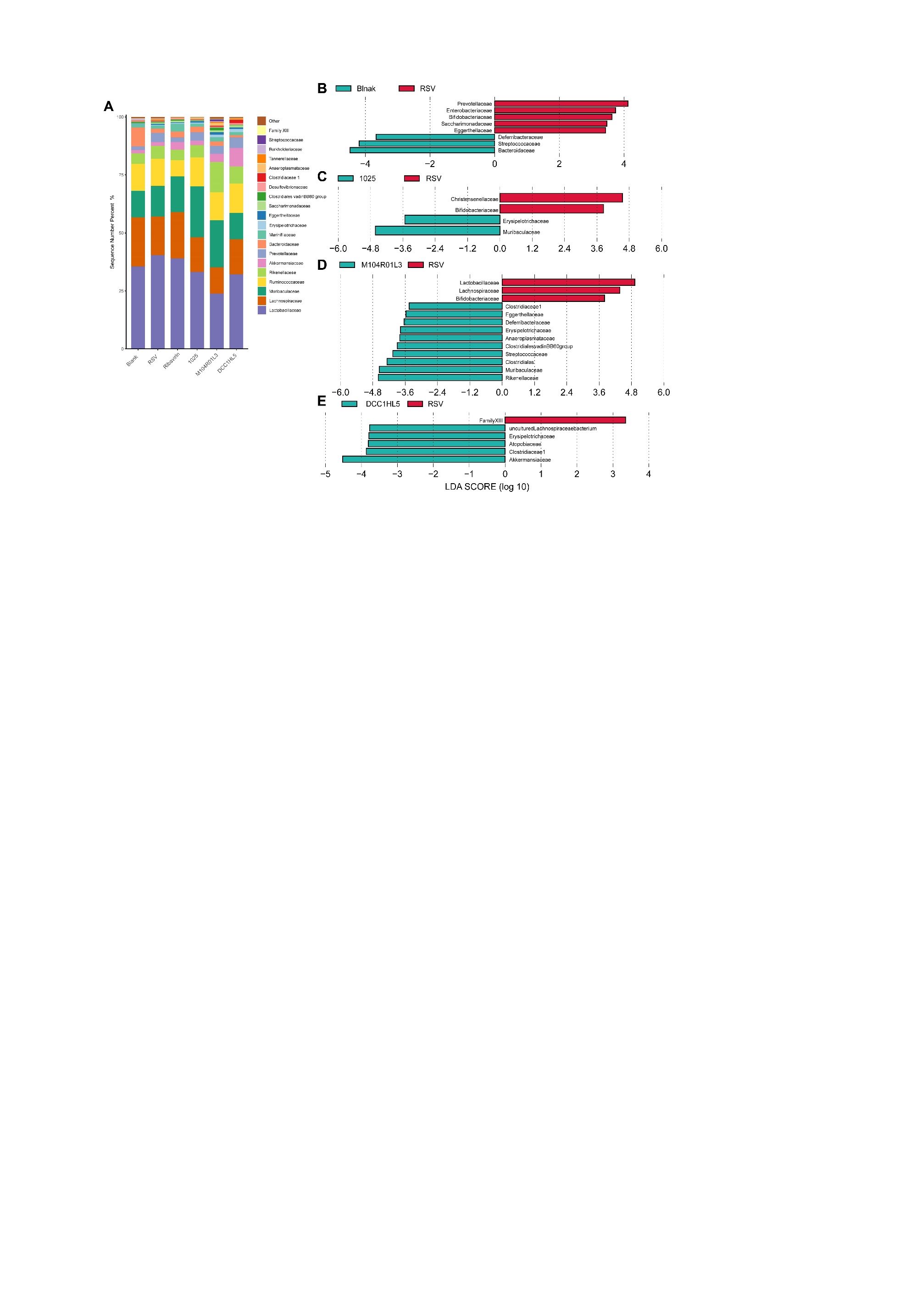


**Supplementary Figure 3|** Impact of three *L. mucosae* strains on the relative abundance of bacterial families.

**(A)** Heatmap analysis of gut microbiota. Linear discriminant analysis effect size (LEfSe) comparison of gut microbes at genus level **(B)** blank and RSV groups, **(C)** 1025 and RSV groups, **(D)** M104R01L3 and RSV groups, and **(E)** DCC1HL5 and RSV groups
